# Effects of Cadmium and Zinc on the Gamete Viability, Fertilization, and Embryonic Development of* Tripneustes gratilla* (Linnaeus)

**DOI:** 10.1155/2016/8175213

**Published:** 2016-04-20

**Authors:** Ivan Patrick B. Tualla, Jayzon G. Bitacura

**Affiliations:** Department of Biological Sciences, Visayas State University, Visca, Baybay City, 6521-A Leyte, Philippines

## Abstract

Heavy metals are frequently reported for their mutagenic and teratogenic effects on benthic organisms. Thus, this study aimed to determine the toxicity of cadmium (Cd) and zinc (Zn) in the gametes of* T. gratilla* and to compare its fertilization and embryonic development under the highest nongametotoxic concentrations of these heavy metals. Gamete viability of* T. gratilla* under CdCl_2_ and ZnSO_4_ treatments was assayed through resazurin reduction test (RRT) and was confirmed through gamete morphology assay. ZnSO_4_ was more toxic to* T. gratilla* gametes than CdCl_2_ and egg cells were more sensitive to both than the sperm cells. Higher concentrations of CdCl_2_ and ZnSO_4_ induced gamete apoptosis and necrosis while highest nongametotoxic concentrations were determined at 1 × 10^−3^ M and 1 × 10^−4^ M, respectively, and were used in an* in vitro* fertilization and embryonic development experiment. ZnSO_4_ treatment inhibited fertilization more than CdCl_2_ and yielded more deformed embryos, while both induced abnormalities and hindered further embryonic development. This study gives the first report on the specific concentrations of Cd and Zn that are toxic to* T. gratilla* gametes and has confirmed the teratogenic effects of these heavy metals.

## 1. Introduction

Heavy metals have been one of the most threatening problems that greatly affect the diversity of life within the marine ecosystem. They are considered as severe pollutants in the natural environment due to their toxicity, bioaccumulation problems [1], and persistence since they remain in the environment for a long period of time [2]. They come from natural sources, such as volcanic basalts [3], and from contamination caused by population growth and industrial development along the coastline communities [4].

Heavy metals could be detected in seawater [5] and sediment [6] and have been found to affect marine organisms even in small concentrations [7, 8]. The harm brought by these pollutants is not only due to the degree of contamination but also due to their biochemical role in the metabolic processes and the extent to which they can be absorbed by marine organisms [2]. Cadmium (Cd) and zinc (Zn) are two of the heavy metals commonly found in the aquatic environment. They are frequently reported to affect water quality and are found to induce mutagenesis and teratogenic effects [9] and decreased abundance and increased mortality of benthic organisms [10, 11].

Along these premises, a study on the effects of heavy metals on marine invertebrates could show that they too are as vulnerable as other marine organisms to these contaminants. Sea urchin, a marine invertebrate, demonstrates a model system for analyzing cellular mechanisms during embryonic development due to their rapid differentiation, and to delineate their critical period of developmental vulnerability [12]. Also, although they have a few number of cell constituents, and they illustrate simple organization [13], their development parallels the same molecular functions observed in higher vertebrates [14].

Although some species of sea urchins have already been studied for the toxic effects of heavy metals on their embryonic development like in the short-spine sea urchin (*Salmacis sphaeroides*),* S. purpuratus*, and* Diadema antillarum*, very few reports have been made on* Tripneustes gratilla*, locally known as* “swaki,”* which is very common in Philippine coastal waters. Most importantly, no reports up to today have really determined the concentrations of heavy metals that could have negative effects on the viability of the gametes of this sea urchin species.

Thus, this study was conducted to determine and compare the toxic effects of Cd and Zn on the gametes of* T. gratilla*, investigate and compare the fertilization efficiencies and the embryonic development of* T. gratilla* under the highest nongametotoxic concentrations of Cd and Zn, and determine and compare the specific morphological abnormalities induced by the highest nongametotoxic concentrations of Cd and Zn on the embryos of* T. gratilla*.

## 2. Materials and Methods

### 2.1. Collection of Samples

A total of 65 adult* T. gratilla* with a diameter ranging from 6.5 to 7 cm were collected from the intertidal zone of Barangay Punta, Baybay City, Leyte. This size range indicates the maturity of sea urchin. Collection of biological samples was done three to four days before full moon of the month since sea urchin follows lunar rhythms [15]. Samples were placed in a large styrofoam container with fresh sea water and immediately transported back to the Department of Biological Sciences, Visayas State University.

### 2.2. Preparation of Acid Wash and Filtered Seawater

All glassware used during induced spawning up to the observation of embryonic development was acid washed. This was done to remove the unwanted heavy metal contaminants that can potentially affect the results and induce bias upon the conduct of the study. Also, prior to the formulation of heavy metal concentrations, seawater was filtered using a system composed of filter flask directly attached to the vacuum pump. A Whatman glass microfiber filter (GF/A) (GE Healthcare Company, UK) with a diameter of 47 mm was added to the filter flask to efficiently filter particles and microorganisms present in the seawater. The filtered seawater was collected into a sterile acid washed container and stored at room temperature.

### 2.3. Induced Spawning of Sea Urchins

Gametes were collected through induced spawning by injecting 0.2 mL of 1 M KCl (Anscom Medical Center, Manila) intracoelomically per 2.5 cm of diameter at the perivisceral cavity near the mouth [16]. Released gametes were identified as either egg or sperm based on its coloration, whitish for males and yellowish for females.

For gamete viability assay, pure concentrations of gametes were collected by directly inverting the sea urchin into sterile acid washed containers, covered with aluminum foil, and were immediately plated for the assays. While for fertilization and embryonic development experiments, the method of Rahman et al. [17] was followed with few modifications. Eggs from female sea urchin were harvested by inverting the sea urchin into a sterile acid washed container containing filtered seawater and were collected at the bottom. On the other hand, “dry” sperm from male sea urchin was collected from the genital pore, transferred into a sterile acid washed container, and covered with aluminum foil. Both egg cells and sperm cells were kept in refrigerator at 4-5°C for not more than 3-4 hrs to maintain its viability.

### 2.4. Formulation of Heavy Metal Concentrations

Cadmium chloride (CdCl_2_) and zinc sulphate (ZnSO_4_) were used for the varying heavy metal concentration treatments. One molar CdCl_2_ (Anscom Medical Center, Manila) and ZnSO_4_ (Ajax Chemical Inc., Australia) were prepared by dissolving their crystals into filtered seawater. From the prepared 1 M solutions, serial dilutions of 0.5 M, 0.1 M, 5 × 10^−2^ M, 1 × 10^−2^ M, 5 × 10^−3^ M, 1 × 10^−3^ M, 5 × 10^−4^ M, and 1 × 10^−4^ M were made.

### 2.5. Gamete Viability Assay

#### 2.5.1. Resazurin Reduction Test (RRT)

RRT was used to test the cytotoxic effects of CdCl_2_ and ZnSO_4_ on* T. gratilla* gametes. It is a convenient reduction test to determine the effects of toxic chemicals in gamete and somatic cells [18–21]. During the assay, sterile 96-well microplates (Corning Inc., USA) were used. Individual wells were designated for each replicate of all the treatments. The treatments were media control (filtered sea water alone), negative control (untreated gametes in filtered sea water), positive control (gametes in filtered sea water treated with 3% hydrogen peroxide [H_2_O_2_]), and the gametes in filtered sea water treated with varying CdCl_2_ and ZnSO_4_ concentrations [T_1_ (1 M), T_2_ (0.5 M), T_3_ (0.1 M), T_4_ (5 × 10^−2^ M), T_5_ (1 × 10^−2^ M), T_6_ (5 × 10^−3^ M), T_7_ (1 × 10^−3^ M), T_8_ (5 × 10^−4^ M), and T_9_ (1 × 10^−4^ M)].

Fifty microliters (50 *μ*L) of concentrated gametes was plated into the wells, except for the wells designated for the media control, and was then added with their respective treatments in a total reaction volume of 100 *μ*L per well. After 4 hrs of incubation 10 *μ*L of resazurin sodium salt solution (Wako Pure Chemical Industries, Ltd., Tokyo, Japan) was added in all the treatment wells. The plates were wrapped with aluminum foil and were incubated at room temperature. After 30 mins, the absorbance of the different treatments at 630 nm was determined using ELx800 Biotek microplate reader at the Biotechnology Laboratory, Visayas State University. This wavelength is where the resazurin has the highest peak and could thus be used to measure the ability of the treated cells to reduce the blue resazurin into pink resorufin constituting an indirect way to measure cell viability.

#### 2.5.2. Gamete Morphology Assay

Another set of treatments was done to observe the morphological effects of varying concentrations of heavy metals on sperm cells and egg cells. The procedure was similar to RRT previously done except that no resazurin solution was added. Gametes were smeared into clean glass slides, air-dried, and immediately fixed using absolute methanol. The slides were then stained with Giemsa and washed with slow running distilled water to remove the excess stain. Once dried, the stained smears were mounted with small drop of Canada balsam (Yana Chemodities, Cebu, Philippines), topped with clean cover slips, warmed to evenly spread the mounting medium, and examined under light microscope.

### 2.6. *In Vitro* Fertilization and Observation of Embryonic Development

#### 2.6.1. Gamete Dilution

Prior to induced fertilization experiment, gametes were diluted first in filtered seawater following the methods described in Edullantes and Galapate [22] and Farley and Levitan [23], with modifications. Both sperm and egg (uncoated) suspensions were incubated at 4°C for almost 30 mins to reduce the metabolism of the gametes and to ensure their viability.

#### 2.6.2. *In Vitro* Fertilization

The highest CdCl_2_ and ZnSO_4_ concentrations tolerable for the gametes particularly to the sperm cells were used in the fertilization experiment following the method of Bielmyer et al. [24] but with few modifications.* In vitro* fertilization was done in separate chambers for the three treatments considered, namely, T_0_ (filtered seawater) this served as the control, T_1_ (CdCl_2_), and T_2_ (ZnSO_4_).

To induce the fertilization of the gametes, 1 mL of egg suspension was added to the beakers containing 9 mL of the treatment solutions. Then, 200 *μ*L of sperm suspension was added to the beakers to start the fertilization. The fertilization chambers were mixed slowly for a minute and were covered with aluminum foil to avoid contamination. These were kept at room temperature.

To compare the fertilization efficiencies of* T. gratilla* under the different treatments, three 20 *μ*L aliquots were obtained for each fertilization chamber after 30 mins. The numbers of fertilized eggs were counted as well as the embryos that are starting to develop. Fertilized egg is characterized with the presence of fertilization membrane. From these data the percent fertilization efficiencies under various treatments were computed using (1). Also counted were unfertilized eggs and data were used in calculating the percent inhibition [22] in various treatments (2)

(1) Percent Fertilization=No. of fertilized eggsTotal no. of eggs×100,

(2) Percent Inhibition=No. of unfertilized eggsTotal no. of eggs×100.

#### 2.6.3. Embryonic Development

The observation of embryonic development was adapted from the method established by Edullantes and Galapate [22]. The observations were made on the following time intervals: 30 mins, 3 hrs, 6 hrs, 9 hrs, 12 hrs, and 24 hrs after the addition of the sperm cells into the chambers.

For each observation time, three 20 *μ*L aliquots of the solution were collected from each treatment and were mounted into a depression slide to observe the embryonic development. Throughout the development, observations were made to record the performance of the embryos under various treatments. The following served as codes for observation: EPC (embryos with pigmented cells), SCD (successful cell division on each cell stage), ESD (embryo stop dividing), NFD (no further development), DE (deformed embryos), ML (malformation of the embryos), and NDAT (no data gathered).

### 2.7. Statistical Analyses

This study followed a completely randomized design (CRD). For the gamete viability assay, analysis of variance (ANOVA) was used to determine the significant difference of the treatments. Post hoc comparison was used to cluster the absorbance of the various treatments following the homogenous subset of Duncan Multiple Range Test. Repeated measures ANOVA with replicated measurements were used to compare the number of deformed embryos on different treatments. All data analyses were carried out using SPSS v.20.

### 2.8. Documentation

Morphology of gametes,* in vitro* fertilization, and embryonic development were documented using a Sony CyberShot digital camera (Sony Inc., Japan) directly focused on a Motic photomicroscope and light microscope (Speed Fair Co. Ltd., Hong Kong).

## 3. Results and Discussion

### 3.1. Cd and Zn Induced Apoptosis and Necrosis in* T. gratilla* Gametes

**Figure 1:**
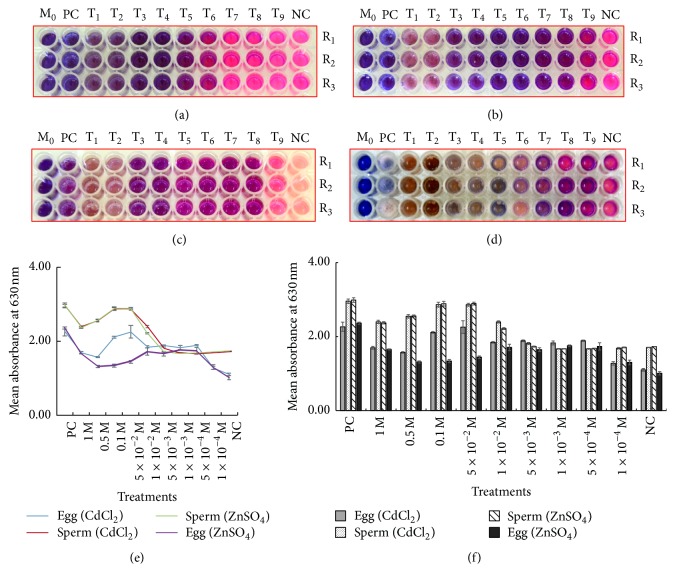
Results of RRT performed on* T. gratilla* sperm cells treated with (a) CdCl_2_ and (b) ZnSO_4_, and egg cells (c and d) treated with the same heavy metal solutions, respectively. M_0_ (media control), PC (positive control), T_1_ (1 M), T_2_ (0.5 M), T_3_ (0.1 M), T_4_(5 × 10^−2^ M), T_5_ (1 × 10^−2^ M), T_6_ (5 × 10^−3^ M), T_7_ (1 × 10^−3^ M), T_8_ (5 × 10^−4^ M), T_9_ (1 × 10^−4^ M), and NC (negative control). R_1_–R_3_ are the replicate wells. (e) Mean absorbance at 630 nm of the wells containing* T. gratilla* gametes with CdCl_2_ and ZnSO_4_ treatments after 30 mins of incubation with resazurin. (f) A histogram showing the mean absorbance at 630 nm of the wells containing* T. gratilla* gametes with CdCl_2_ and ZnSO_4_ treatments after 30 mins of incubation with resazurin.

Results of the resazurin reduction test for the cytotoxic effects of CdCl_2_ and ZnSO_4_ in* T. gratilla* gametes are shown in Figure 1. Color changes differed between the sperm cells and the egg cells and among the heavy metal dilution series. The reduction of resazurin to resorufin is manifested by changes in color from blue to pink as observed in sperm cells treated with 1 × 10^−3^ M to 1 × 10^−4^ M (T_7_–T_9_) CdCl_2_ and 1 × 10^−4^ M (T_9_) ZnSO_4_. For the egg cells, color changes were only evident in those treated with 1 × 10^−4^ M CdCl_2_ and slight changes in the same concentration in ZnSO_4_ treatment. These color changes (from blue to pink) are similar to the untreated egg cells (negative control) which implies that at these concentrations the cells are still viable because they were still able to reduce resazurin to resorufin. These results could only be attributed to the CdCl_2_ and ZnSO_4_ treatments because media control showed no color change, implying that the medium (filtered seawater) contained no other cell that could reduce resazurin to resorufin. On the other hand, there was no pink coloration observed in sperm cells treated with 1 M down to 5 × 10^−3^ M CdCl_2_ and 1 M to 5 × 10^−4^ M ZnSO_4_. The same for egg cells treated with 1 M to 5 × 10^−4^ M CdCl_2_ and 1 M to 1 × 10^−4^ M ZnSO_4_. The same result was observed in positive control indicating the cytotoxic effects of these CdCl_2_ and ZnSO_4_ concentrations to* T. gratilla* gametes.

Furthermore, percent reduction of resazurin to resorufin was supposed to be computed in all treatment. It was not done for several reasons. First, the microplate reader does not have the wavelength necessary for detecting the absorbance of resorufin (540–570 nm) as needed in the calculation. Secondly, resazurin did not completely mix with the cells on higher concentrations of CdCl_2_ and ZnSO_4_ treatments making the lower lethal treatments bluer than expected. However, the microplate reader has available wavelength for resazurin ranging from 600 to 630 nm with the peak at the latter. Since it was necessary to compare the treatments quantitatively, it was decided to do it using treatment absorbance at 630 nm. This quantified the amount of resazurin that is present in the treatment wells. This means that the more viable the cells are, the more they are able to reduce blue resazurin to pink resorufin giving low absorbance at 630 nm. While the treatments are more toxic, the ability of the cells to reduce resazurin to resorufin will be lesser giving higher absorbance at 630 nm. This is with the exception of course of the treatments where cells were suspected to be necrotic resulting for the resazurin to be immiscible because of the viscous media. This would mean that, hypothetically, highest absorbance will be acquired at the treatments where cells are still in the apoptotic phase of cell death and would start to drop at the necrotic phase caused by the higher concentrations of CdCl_2_ and ZnSO_4_ treatments. Moreover, the intensity of pink coloration between the untreated (negative control) egg cells (Figures 1(c) and 1(d)) and untreated sperm cells (Figures 1(a) and 1(b)) was due to its natural yellowish coloration while the latter has a whitish coloration. Both untreated gametes later on indicated the reduction of the blue resazurin. Resazurin can be further reduced into white dihydroresorufin [18] which was demonstrated by the untreated egg cells (negative control) in Figure 1(c).  Figure 1(e) shows the mean absorbance reading of the gametes treated with CdCl_2_ and ZnSO_4_ as compared to the positive and negative controls. Media controls were not included because they have no cells in them giving different absorbance values. These results supported the earlier hypothesis. The absorbance readings of the gametes treated with CdCl_2_ and ZnSO_4_ have similar trends. Lowest absorbance was obtained at treatments with no cytotoxic effects and increased absorbance was obtained as the cells started to die. Highest (peak) absorbance implied the highest concentration that induced apoptosis on the cells and then decreased when cells became necrotic.

Moreover, analysis of variance (ANOVA) revealed highly significant difference between the different CdCl_2_ and ZnSO_4_ treatments on both sperm cells and egg cells. Absorbances were further homogenized using DMRT for both CdCl_2_ and ZnSO_4_ and were clustered according to the closeness of value to each other. Importance was given on determining which concentrations were not significantly different to the negative control since these concentrations were most probably the ones having no toxic effects on the gametes.

To disregard the effect of chloride and sulphate constituents of Cd and Zn, another set of RRT was conducted on the gametes. The Cl_2_ and SO_4_ compounds with nonheavy metal constituent were used. These were NaCl and Na_2_SO_4_. Results (pictures not shown) revealed that there was a significant difference between the effects of the same molar concentrations of CdCl_2_ and ZnSO_4_ and NaCl and Na_2_SO_4_ on* T. gratilla* gametes. NaCl and Na_2_SO_4_ treatments did not induce apoptosis and necrosis on the cells as compared to the effects of CdCl_2_ and ZnSO_4_ treatments. This implies that the chloride and sulphate components of CdCl_2_ and ZnSO_4_ have no toxic effects on* T. gratilla *gametes, only Cd and Zn.

The variation of the absorbance among the different treatments of CdCl_2_ and ZnSO_4_ is influenced by the capacity of the gametes to maintain metabolic activity [25] and in worst case by greatly affecting the gamete's morphology [26]. Due to the minimal availability of advance molecular stains and equipment especially in developing countries, Giemsa staining was conducted since it is needed to validate the prior suspicion that the gametes underwent apoptosis and necrosis under CdCl_2_ and ZnSO_4_ treatments. The morphology assay was conducted through Giemsa staining which caused the blue coloration of the gametes due to the attachment of dye cations (azure B and methylene blue) present in the Giemsa mixture onto the phosphate anions of DNA supported by van der Waals forces in sites where adenine and thymine are found rich in nature [27].

**Figure 2:**
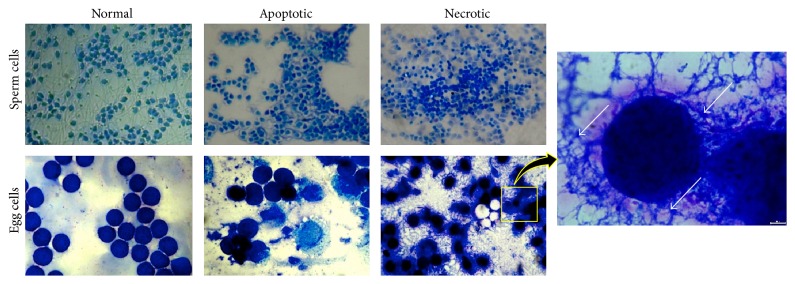
Representative images showing* T. gratilla* different gamete morphologies (400x). This network of thread-like blue material (white arrows) strongly indicates that there is a result of leaching DNA materials when a cell undergoes necrosis due to the attachment of Giemsa stain onto sites where adenine and thymine are rich.

Results revealed that both egg cells and sperm cells treated with CdCl_2_ and ZnSO_4_ underwent apoptosis and necrosis proving the earlier suspicion (Figure 2). Saikumar and Venkatachalam [28] described necrotic cell as a result of lethal or accidental actions by toxins or physical cause [29]. It can be observed by cellular edema [30], cell shrinkage [31], dissolution of nuclear chromatin, disruption of plasma membrane, and release of intracellular contents into the extracellular space [28]. Similarly, Migliarini et al. [32] reported that cell exposed to high concentration of toxins will undergo necrosis and suggested that cell treated with minimal amount of toxins will undergo apoptosis. Furthermore, apoptosis is characterized by cellular morphological changes and formation of membrane-bound cell fragments called apoptotic bodies [33], blabbing of plasma membrane and nuclear degeneration [32], and DNA laddering and chromatin condensation [29].

Table 1 shows the summary of the gamete viability test. Highest nongametotoxic concentrations of heavy metals were identified, 1 × 10^−3^ M for CdCl_2_ and 1 × 10^−4^ M for ZnSO_4_. These were the concentrations at which no significant difference was shown as compared to the negative control. And most importantly, these concentrations gave clear negative cytotoxicity results on sperm cells as revealed through RRT. These two concentrations were used for the* in vitro* fertilization and embryonic development of* T. gratilla* since sperms are most likely to fertilize mature egg cells at these levels.

### 3.2. Highest Nongametotoxic Concentrations of Cd and Zn Reduced Fertilization Efficiency of* T. gratilla* Gametes

**Figure 3:**
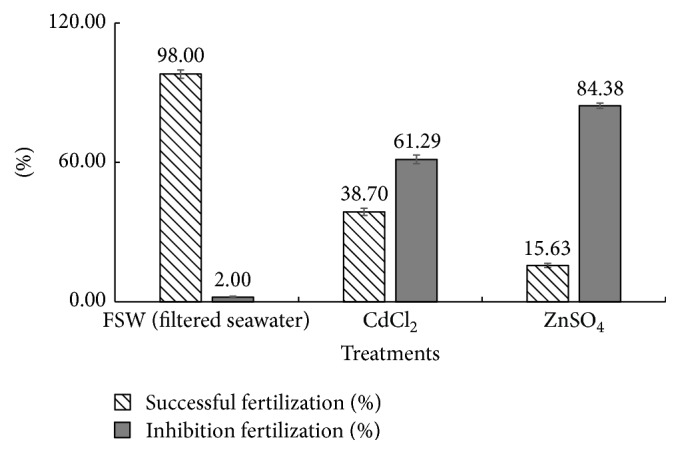
The percent successful fertilization and inhibition of fertilization as observed on the gametes after 30 mins exposure to 1 × 10^−3^ M CdCl_2_, 1 × 10^−4^ M ZnSO_4_ and filtered seawater.

Figure 3 shows significant differences (*p* < 0.05) in percent successful fertilization and inhibition of fertilization among the different treatments. Gametes exposed to 1 × 10^−3^ M CdCl_2_ and 1 × 10^−4^ M ZnSO_4_ have ~3-fold and ~6-fold lower percentages of successful fertilizations as compared to the control (only filtered seawater), respectively. These imply that ZnSO_4_ treatment has inhibited a higher number of fertilizations as compared to CdCl_2_ treatment.

A study conducted by Gopalakrishnan et al. [34] showed that the spermatozoa of* Hydroides elegans *are sensitive to Cd and Zn and reduced their fertilization rate. In addition, Edullantes and Galapate [22] reported the toxicity of Zn to* T. gratilla* sperm cells and that it induced the inhibition of the fertilization. Moreover, Cd has been reported to diminish the glucose utilization and oxidation in spermatozoa that affects sperm motility while Zn affects sperm motility by mediating a common cation-binding site found in adenyl cyclase (as cited by Ebrahimi [25]). Furthermore, Zn was reported to interfere with the cortical granule-derived protease that blocks the formation of fertilization membrane (as cited by Edullantes and Galapate [22]) and Cd ions were reported to have a great influence in blocking Ca^2+^ in spermatozoa (as cited by Ebrahimi [25]).

### 3.3. Highest Nongametotoxic Concentrations of Cd and Zn Caused Abnormalities in the Development of* T. gratilla* Embryos

**Figure 4:**
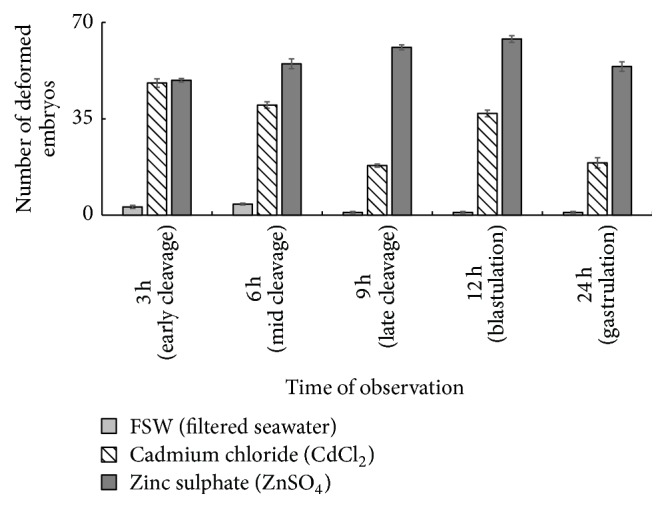
Number of deformed embryos observed under the three treatments considered starting from early cleavage up to 2-arm echinopluteus.

As the time of observation increased, ZnSO_4_ yielded more deformed embryos than CdCl_2_ as shown in Figure 4. Analysis of variance (ANOVA) revealed that the number of deformed embryos treated with nongametotoxic concentrations of CdCl_2_ and ZnSO_4_ that were observed throughout the embryonic development of* T. gratilla* significantly differed. Along with this, highest nongametotoxic concentration of ZnSO_4_ resulted in 19 deformed embryos compared to CdCl_2_ indicating that Zn is more toxic than Cd. A study conducted by Xu et al. [35] found that Zn was more toxic than Cd in the embryos of* Strongylocentrotus intermedius*.

**Figure 5:**
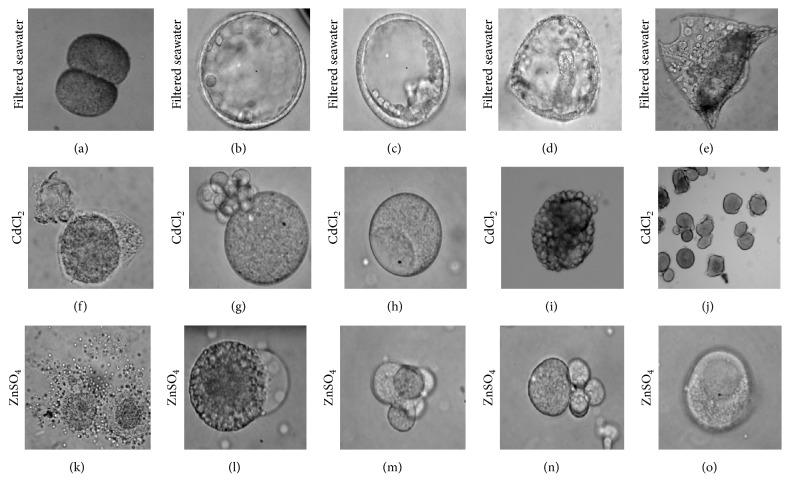
The embryonic development of* T. gratilla.* Normal development observed starting from 2-cell up to 2-arm echinopluteus on the control (filtered seawater; a–e) and abnormalities in the embryos observed under the highest nongametotoxic concentrations of CdCl_2_ (f–j) and ZnSO_4_ (k–o).

Table 2 summarizes the general observations of the embryos treated with CdCl_2_ and ZnSO_4_. The results showed that these two nongametotoxic concentrations can inhibit the embryonic development at early stages of cleavage. As the exposure time of the embryos increased these heavy metals eventually arrested the embryonic development of* T. gratilla *at blastula leaving the control (FSW) embryos to develop into a normal echinopluteus. Kobayashi [36] reported that in several species of sea urchins, blastula appears to be also sensitive to toxic substances following the gastrula and fertilization. Figures 5(a)–5(e) show the normal development of embryos under filtered seawater (FSW). Normal development of the embryos was observed following the observation time starting from 2-cell stage up to the formation of 2-armed echinopluteus.

Moreover, the embryos treated with the highest nongametotoxic concentration of CdCl_2_ were observed to be deformed throughout the observation time as shown in Figures 5(f)–5(j). The following abnormalities were observed on the embryos treated with CdCl_2_, namely, cytolysis (Figure 5(f)), cell fragmentation (Figure 5(i)), presence of apoptotic bodies (Figure 5(g)), and blebbing of embryos that was observed in the unsuccessful cell division at 2-cell stage (Figure 5(j)). The highest nongametotoxic concentration of ZnSO_4_ also induced deformities such as blebbing of embryos, cytolysis, and unequal cell division (Figures 5(k)–5(o)).

A study conducted by Migliarini et al. [32] reported that Cd can induce the expression of caspase-3 gene and 70 kDa heat shock protein (HSP70) that serves as biochemical marker when cells are exposed to environmental stresses such as exposure to toxic substances. Also, Warnau et al. [37] reported that Cd induced severe skeletal malformation in the embryos that contributes to the embryotoxicity of heavy metals.

Zn affects embryos through the disruption of ribosomal RNA synthesis and inhibits the development of endoderm and mesenchyme derivatives resulting in abnormalities (Pirrone et al. 1970, Timourian 1968, as cited by Edullantes and Galapate [22]). The embryotoxicity of heavy metals such as Cd and Zn can be reflected due to the limited capacity of metallothioneins (MTs), a group of low molecular mass proteins that has high affinity to heavy metals, to bind onto these contaminants in the environment [37].

Voronina and Wessel [38] reported that blebbing of sea urchin embryos due to apoptosis resulted in morphological changes, chromatin degradation, and activation of caspases. Moreover, Sasaki and Chiba [33] reported that fragmentation is caused by the reorganization of F-actin that regulates membrane and fragmentation of the embryos which follows either one of the two pathways, namely, extrinsic pathway, which is described as the death of cells using a death receptor and during egg cell death which put an emphasis that caspase-3 proteases regulate the death of the sea stars' eggs. Furthermore, apoptosis of cells follows intrinsic pathway or also known as the mitochondrial pathway [29].

The highest nongametotoxic concentration of CdCl_2_ and ZnSO_4_ used for the* in vitro* fertilization and embryonic development is equivalent to 1.8 *μ*g·L^−1^ and 2.8 *μ*g·L^−1^, respectively. Allowable concentration of Cd effluents from industrial factories should not exceed 100–200 *μ*g·L^−1^ according to DENR [39]. In this study, the concentration used for CdCl_2_ is ~52-fold less than the allowable amount of Cd that is set by the DENR which means that* T. gratilla* is very vulnerable and sensitive to the allowable amount of Cd effluents in the marine environment.

Furthermore, the amount of Cd detected in seawater near the industrial sites ranges from 0.002 *μ*g·L^−1^ to 4.3 *μ*g·L^−1^ reported by Kobayashi and Okamura [40]. Comparing the maximum amount of Cd detected in industrial waters to the amount used in the embryotoxicity assay, the latter is 2-fold lower and yet abnormalities on the embryos are still prominent and had eventually arrested the embryonic development of* T. gratilla*. In addition, Warnau et al. [37] reported that the lowest embryotoxic concentration of CdCl_2_ for* Paracentrotus lividus* is 10^−5^ M which is lower than the 10^−3^ M reported in this study.

Conversely, no available information was gathered on the amount of Zn effluents allowed by DENR; however, several studies suggest that the tolerable amount of Zn in the marine environment should not exceed 4.9 *μ*g·L^−1^–5.0 *μ*g·L^−1^ [40, 41]. David [42] reported that the highest detected level of Zn in the sediment of the Philippines, where mine-tailing spills are unloading effluents, is 2.76 *μ*g·L^−1^. This is ~1-fold lower than the level of Zn used in this study. The study conducted by David [4] was conducted 8 years ago and the amount of Zn that can be detected in Philippine waters with the presence of industrial factories might be higher compared to today.

The performance of embryos treated with 2.8 *μ*g·L^−1^ in this study shows that even in almost ~1-fold lower than the tolerable Zn input in the marine environment abnormalities on* T. gratilla* gametes are still evident. Comparatively, Edullantes and Galapate [22] reported that embryonic development of* T. gratilla* is concentration-dependent and stage-specific inhibition starting from fertilization to blastulation ranges from 42 to 93 *μ*g·L^−1^. On the other hand, Kobayashi and Okamura [40] reported that Zn has no inhibitory effects on the embryos of* Anthocidaris crassispina* at 7.2 *μ*g·L^−1^. The sampling site of the study is one of the marine protected areas in the vicinity of Baybay City, Leyte, where anthropogenic activities are strictly monitored. That includes the prevention of industrial factories to be established near coastal area that can induce heavy metal contaminants. Through this strong monitoring by the local government unit, this study indicated that the coastal area of Barangay Punta still harbors the most negligible possible amount of heavy metals such as Cd and Zn.

## 4. Conclusions

Following a unique experimental approach aimed at providing a more realistic context of an environmental milieu, this study demonstrated that Cd and Zn have toxic effects on the gametes of* T. gratilla* with egg cells being more sensitive than the sperm cells. This study also gave the first report on the specific concentrations that induce apoptosis and necrosis on the gametes of this sea urchin species. Moreover, even the concentrations that are nontoxic to their gametes have significantly reduced the fertilization efficiency of this sea urchin species and have caused abnormalities on their embryos and arrested the embryonic development. Specific embryonic abnormalities were also reported.

These results have huge impact in the formulation of environmental policies for the regulation of the amount of the heavy metals Cd and Zn in industrial effluents. This is especially because the current policy on the allowable amount of Cd imposed by the DENR regulated industrial effluents at disturbingly higher levels than what was being shown as toxic in this study. Conversely, no policy concerning the allowable amount of Zn in industrial effluents is currently available in the Philippines.

## Figures and Tables

**Table 1:** Summary of viability assay on *T. gratilla* gametes treated with CdCl_2_ and ZnSO_4_.

	CdCl_2_		ZnSO_4_	
	Sperm	Egg	Sperm	Egg
Normal	1 × 10^−4^ M–1 × 10^−3^ M^*∗*^	1 × 10^−4^ M	1 × 10^−4^ M^*∗*^	1 × 10^−4^ M–5 × 10^−4^ M
Apoptosis	5 × 10^−3^ M–0.1 M	5 × 10^−4^ M–0.1 M	5 × 10^−4^ M–0.1 M	1 × 10^−3^ M–0.1 M
Necrosis	0.5 M–1 M	0.5 M–1 M	0.5 M–1 M	0.5 M–1 M

^**∗**^Highest nongametotoxic concentrations.

**Table 2:** The morphological observations on the embryos of *T. gratilla* treated with FSW, highest nongametotoxic concentration of CdCl_2_ and ZnSO_4_ during 3 h, 6 h, 9 h, 12 h, and 24 h.

Time of observation		Treatments	
	FSW (filtered seawater)	Cadmium chloride (CdCl_2_)	Zinc sulphate (ZnSO_4_)
3 hrs			
2-cell	SCD	SCD	SCD
4-cell	SCD	SCD	SCD
6 hrs			
8-cell to 16-cell	SCD	SCD, DE, EPC	SCD, DE, EPC
9 hrs			
32-cell to 64-cell	SCD	ESD, DE	ESD, DE
12 hrs			
Blastula	SCD	NFD, DE	NFD, DE
24 hrs			
Gastrula	SCD	NFD	NFD
2-arm echinopluteus	SCD	NFD	NFD
General observation	No abnormalities	Formation of apoptotic bodies, cell fragmentation	Blebbing and deformed embryos

EPC = embryos with pigmented cells, SCD = successful cell division, ESD = embryo stop dividing, NFD = no further development, and DE = deformed embryos.
